# Cryo-Milled β-Glucan Nanoparticles for Oral Drug Delivery

**DOI:** 10.3390/pharmaceutics16040546

**Published:** 2024-04-16

**Authors:** Guanyu Chen, Yi Liu, Darren Svirskis, Hongyu Li, Man Ying, Weiyue Lu, Jingyuan Wen

**Affiliations:** 1 School of Pharmaceutical Sciences (Shenzhen), Shenzhen Campus of Sun Yat-sen University, Shenzhen 518107, China; 2 School of Pharmacy, Faculty of Medical and Health Sciences, University of Auckland, 95 Park Road, Grafton, Auckland 1142, New Zealand; 3 School of Medicine, University of Texas Health San Antonio & College of Pharmacy, University of Texas, Austin, TX 78759, USA; 4 Department of Pharmaceutics, School of Pharmacy, Zhangjiang Campus of Fudan University, 826 Zhangheng Road, Pudong New Area, Shanghai 200433, China

**Keywords:** β-glucan, cryo-mill, cryogenic, nanoparticle, sustained release

## Abstract

Gemcitabine is a nucleoside analog effective against a number of cancers. However, it has an oral bioavailability of less than 10%, due to its high hydrophilicity and low permeability through the intestinal epithelium. Therefore, the aim of this project was to develop a novel nanoparticulate drug delivery system for the oral delivery of gemcitabine to improve its oral bioavailability. In this study, gemcitabine-loaded β-glucan NPs were fabricated using a film-casting method followed by a freezer-milling technique. As a result, the NPs showed a small particle size of 447.6 ± 14.2 nm, and a high drug entrapment efficiency of 64.3 ± 2.1%. By encapsulating gemcitabine into β-glucan NPs, a sustained drug release profile was obtained, and the anomalous diffusion release mechanism was analyzed, indicating that the drug release was governed by diffusion through the NP matrix as well as matrix erosion. The drug-loaded NPs had a greater ex vivo drug permeation through the porcine intestinal epithelial membrane compared to the plain drug solution. Cytotoxicity studies showed a safety profile of the β-glucan polymers, and the IC_50s_ of drug solution and drug-loaded β-glucan NPs were calculated as 228.8 ± 31.2 ng·mL^−1^ and 306.1 ± 46.3 ng·mL^−1^, respectively. Additionally, the LD_50_ of BALB/c nude mice was determined as 204.17 mg/kg in the acute toxicity studies. Notably, pharmacokinetic studies showed that drug-loaded β-glucan NPs could achieve a 7.4-fold longer T_1/2_ and a 5.1-fold increase in oral bioavailability compared with plain drug solution. Finally, in vivo pharmacodynamic studies showed the promising capability of gemcitabine-loaded β-glucan NPs to inhibit the 4T1 breast tumor growth, with a 3.04- and 1.74-fold reduction compared to the untreated control and drug solution groups, respectively. In conclusion, the presented freezer-milled β-glucan NP system is a suitable drug delivery method for the oral delivery of gemcitabine and demonstrates a promising potential platform for oral chemotherapy.

## 1. Introduction

Cancer is still one of the major causes of death, and its incidence is continuously increasing. Most anticancer drugs are generally administered through intravenous (*i.v*.) injection or infusions, and this often causes the administered drug to exceed the maximum tolerable concentration, which leads to serious side effects and subsequent fast excretion from the circulation system, resulting in low oral drug bioavailability [1].

Among all the drug administration routes, the oral route is the most preferred for most drug delivery due to greater patient compliance, lower production costs, and higher therapeutic efficacy [2,3]. Oral chemotherapy is usually designed with a sustained drug release form and thus is able to maintain a moderate plasma drug concentration over a period of time to achieve a prolonged exposure of tumors to the drug as well as to avoid a drug dose over the maximum tolerable dosage, resulting in much higher drug efficacy without side effects [4]. However, a large number of orally administered anticancer/cytosine drugs have difficulties entering the systemic circulation due to the poor solubility, permeability, and stability of the drugs. In addition, other associated challenges include hepatic first-pass metabolism, intestinal enzymatic degradation, and P-glycoprotein (P-gp) efflux [5].

Polymeric nanoparticles (NPs) with a small particle size of less than 1 µm have raised interest as a drug carrier system for a wide range of drug candidates. It offers advantages over other colloidal carriers in terms of greater drug stability in the gastrointestinal (G.I.) tract and greater ability to minimize its intestinal enzymatic degradation and P-gp efflux in general [6,7]. It has the potential to improve drug permeability, with its sustained drug release manner, resulting in higher oral drug bioavailability, thus promoting the therapeutic effect [8,9]. In addition, NPs have a smaller particle size than microparticles, which allows for enhanced extravasation into tumor sites by the enhanced permeability and retention (EPR) effect [10].

β-Glucans are linear, unbranched polysaccharides containing monomeric β-D-glucopyranosyl residues with linkages of (1-3)-O-linked-β-D-glucopyranosyl units and (1-4)-O-linked β-D-glucopyranosyl units [11]. They have attracted scientists’ attention and have been utilized to fabricate oral pharmaceutical formulations since formulations using β-glucans can ensure (1) the prolonged release of the drug candidate; (2) great protection from hydrolytic and enzymatic degradation, thus resulting in longer drug half-life; and (3) the stimulation of the immune response, which is beneficial for some immune-related drugs [12]. Cyclodextrins have been recently receiving growing attention since they have complex-forming ability with drug candidates and are able to enhance the solubility of hardly soluble drugs. However, the rigid structure and poor water solubility of cyclodextrins limit their further application. Compared with cyclodextrins, the advantages of β-glucans over cyclodextrins mainly involve their higher water solubility; additionally, β-glucans also exhibit high viscosities at very low concentrations (1%) and are stable with a wide range of pH levels [13]. Peter et al. reported that the oral delivery of β-glucan was bound by intestinal epithelial and gut-associated lymphoid tissue (GALT) cells and pointed to the promotion of the systemic levels of the drug candidate [14]. Huong et al. encapsulated curcumin into β-glucan NPs (Cur–Glu) and found them to be much more soluble in water not only compared with free curcumin but also compared with other biodegradable polymer-encapsulated curcumin NPs [15].

Milling is a common mechanical process to reduce the particle size of NPs, and it has been frequently used in the pharmaceutical industry [16]. However, the high levels of mechanical energy used during milling may cause particle fracture and changes in crystal structure of drugs, including polymorphic transformations, as well as localized heating, which may result in drug degradation [17]. In contrast, cryo-milling is a high-energy milling process operated at a very low temperature using cryogenic media such as liquid nitrogen [18]. This method represents an effective technique for reducing the risk of the recrystallization of the amorphous material due to reduced heat production compared to conventional continuous milling. Freezer mills consist of a compartment containing liquid nitrogen (−195.5 °C) that cools down the entire internal assembly, which consists a polycarbonate tube containing the sample and a steel impactor for milling the sample. The sample is embrittled with liquid nitrogen and then milled with the steel impactor, which is magnetically driven back and forth against the stationary end plugs. According to the machine specification, the procedure includes precooling, milling, and cooling in between each cycle, and the impact rate can be adjusted. In this study, the optimal milling settings of the program were investigated. The advantage of this technique is its low cost, easy fabrication, and great availability for scale-up production.

Gemcitabine (2′, 2′difluorodeoxycytidine), is a well-known anticancer drug that is currently in clinical use for the treatment of several types of cancers. Unfortunately, it is rapidly metabolized with a short plasma half-life of 8–17 min via intravenous infusion [19], and its cytostatic action is strongly exposure-time-dependent. It is rapidly deaminated by cytidine deaminase in the blood, kidney, and liver [20]. Thus, in order to maintain a required plasma concentration over a sufficient period, repeated administration of relatively high doses is required. However, this will lead to severe systemic toxicity [21]. 

To date, there has been no oral formulation of gemcitabine in the market. Additionally, this is the first time to report this novel health-beneficial carrier system for oral gemcitabine delivery. β-Glucan is a well-known nutrient that is beneficial for gastrointestinal health as well as the overall immune system. Therefore, this is an environmentally friendly oral delivery system and a promising oral drug carrier as well as a nutrient beneficial for human health. Secondly, the freezer-milled technique is of benefit to pharmaceutical researchers, since it has significant advantages such as ease in processing capable of scale-up and low cost, thus demonstrating its great potential in future applications involving the development of nanomedicines [22].

## 2. Materials and Methods

### 2.1. Materials

Gemcitabine hydrochloride, MW 299.66, β-glucan, (hydroxypropyl)methyl cellulose (HPMC), ethylcellulose (with 48% ethoxy content) (EC), acetic acid, 3-(4, 5-dimethyl-thiazol-2-yl)-2, and 5-diphenyl tetrazolium bromide (MTT) were purchased from Sigma-Aldrich (St. Louise, MO, USA). Methylcellulose LR was purchased from Chem-Supply (Gillman, South Australia). Chitosan (MW 150,000, DD 70%), was purchased from Comwin Fine Chemicals Co. (Changzhou, China). Methylcellulose (MC) was purchased from ICN Biomedical, Inc. (Santa Ana, CA, USA). Polypropylene glycol was purchased from Midwest Pharmaceutics (Hawke’s Bay, New Zealand). Dimethyl sulfoxide (DMSO) was purchased from Labpartner (Shanghai, China). The 4T1breast cancer cell line was purchased from the American Type Culture Collection (ATCC; Rockville, MD, USA). The Roswell Park Memorial Institute (RPMI) medium 1640 basic was purchased from Gibco (Grand Island, NE, USA), and fetal calf serum was purchased from Hyclone (Logan, UT, USA). Penicillin–streptomycin–glutamine and nonessential amino acids were purchased from Life Technology (Grand Island, NY, USA). All other chemicals used were of reagent grade. Milli-Q water was obtained through reverse osmosis using a Millipak^®^ system (Millipore, Burlington, MA, USA, 0.22 µm). 

### 2.2. Methods

The selected polymers and gemcitabine were mixed with various concentrations, and the films were developed using the conventional casting method [23]. Briefly, 15 mL of different concentrations of water-soluble mucoadhesive polymers (MC, HPMC, EC, β-glucan, and chitosan) were individually mixed with gemcitabine (0.5% *w*/*v*), and 5 mL of 5% *v*/*v* polypropylene glycol was added as a plasticizer. The solution was mixed well and the resulting gel-like solution was then cast on a Petri dish and dried in an oven at 37 °C for 48 h. Once the films had formed, they were trimmed into small pieces and loaded into the milling vials containing an impactor. Subsequently, an intensive milling process was carried out via a freezer mill (6970EFM Freezer/Mill^®^, Metuchen, NJ, USA) with optimized settings to generate pulverized drug-loaded NPs. The optimal formulation was selected from characterization and carried out for cellular uptake and transport studies, in vivo pharmacokinetic studies, and pharmacodynamic studies.

## 3. Characterization 

### 3.1. Optimization of Polymeric Films and Freezer Mill Process Settings

MC, HPMC, EC, β-glucan, and chitosan were selected for forming the polymeric films. The first step of this process involved finding suitable polymers to form the films using the casting method. Meanwhile, suitable concentrations of the polymers to produce the films with the ideal physical appearance and stability were investigated. The optimal films were selected to carry out the milling process. Different settings of precooling time, milling period, operating cycles, and impact rate were trialed. The optimal setting was predicted from the resultant NPs with the optimal physical appearance and relatively smaller particle size.

### 3.2. Particle Size, Zeta Potential, Surface Morphology, and Entrapment Efficiency

The particle size, Polydispersity Index (PDI), and zeta potential of the cyro-milled NPs were determined using a Malvern Zetasizer Nano-ZS (Malvern Instruments Ltd., Malvern, UK). The surface morphology of the NPs was observed by using scanning electron microscopy (SEM, Phillips XL305 Field Emission Gun, Eindhoven, The Netherlands). The obtained NPs in powder form were sputter-coated with a thin layer of platinum on the surface, and images were captured using SEM. The drug entrapment efficiency (EE) was determined using a reverse-phase high-performance liquid chromatography (HPLC) method indirectly [24]. A GraceSmart C18 column (250 × 4.6 mm, 5 µm; GraceSmart, India) was used, in which acetonitrile and 1.38% *w*/*v* pH 6.5 sodium dihydrogen phosphate buffer at the volume ratio of 7:93 were added in the mobile phase, and the parameters were set as 1 mL/min flow rate and 270 nm UV detection [25].

### 3.3. In Vitro Drug Release Studies

The in vitro release of gemcitabine from NPs was studied using a Franz diffusion apparatus (FDC-6, Logan, NJ, USA). The gemcitabine solution (1% *w*/*v*) and an equivalent amount of the drug comprising gemcitabine-loaded NP samples were added to the donor chamber, with a cellulose membrane (MW 12,000, Membra-Cel^®^, Viskase, Loudon, TN, USA) sandwiched between the donor and receptor chambers, maintaining the sink condition. PBS with pH 7.4 was used as the medium, and the temperature was kept at 37 ± 1 °C. Then, aliquots of 500 μL each were withdrawn at various time intervals, and 500 μL of fresh PBS was replaced. The release drug amount over time was determined with HPLC. 

The release kinetics were evaluated using four different models, namely zero order, first order, Higuchi equation, and Korsmeyer–Peppas equation, to characterize the mechanism of drug release from the NPs. For the different models, the obtained data were plotted in the following manner: the zero-order model as the cumulative percentage of drug release versus time, the first-order model as the log cumulative percentage of drug remaining versus time, Higuchi’s model as the cumulative percentage of drug released versus square root of time, and the Korsmeyer–Peppas model as log the cumulative percentage of drug released versus log time.

### 3.4. Ex Vivo Permeation Studies 

The ex vivo permeation study of gemcitabine-loaded NPs was also conducted using Franz diffusion cells with the same method as that used in the drug release study. Here, the cellulose membrane was replaced with the porcine intestinal epithelial membrane. Porcine mucosal tissue was previously soaked in pH 7.4 PBS overnight. After the accumulative drug amount was determined using HPLC, the apparent permeation coefficient (P_app_) was calculated. P_app_ is the rate at which a drug will permeate over the intestinal wall and enter the portal circulation [26], and it is calculated using Equation (1):

(1) $$P_{\mathrm{app}}=\frac{dXr}{dt}\times \frac{1}{A\cdot Co}$$

where Xr is the cumulative drug amount (µg), A is the area of the permeation membrane (cm^2^), C_0_ is the initial concentration in the donor compartment (µg/mL), and dX_r_/dt is the flux, which is the gradient of the best-fit line when the cumulative amount of drug permeated over time [27].

### 3.5. Cell Culture and Cytotoxicity Studies

Briefly, 4T1, a breast cancer cell line derived from a BALB/c mammary tumor, was cultured in an RPMI medium 1640 basic containing 10% fetal calf serum, 1% penicillin–streptomycin–glutamine, and 1% nonessential amino acids. The cells were cultured as a monolayer at 37 °C in a 98% humidified atmosphere containing 5% CO_2_.

The cytotoxicity of the NP materials was evaluated using an MTT assay. The cells were cultured on 96-well microtiter plates (0.1 mL, containing approximately 5 × 10^3^ cells/well) for 24 h at 37 °C to allow cell attachment to the plates. Briefly, 100 µL of the drug solution and drug-loaded NPs with various concentrations (established via serial dilution) were added to the growth medium and incubated for another 72 h under the same conditions. To evaluate cell survival, 20 µL of the 5 mg/mL MTT solution was added to each well and incubated for 4 h. Subsequently, the MTT solution was withdrawn, and 200 µL of dimethyl sulfoxide (DMSO) was added to dissolve formazan crystals; then, the absorbance was measured with a microtiter plate reader (SpectraMax^®^ Plus, Molecular Devices, San Jose, CA, USA) at 596 nm. The percentage cell viability was calculated using the absorbance test, and the values of treated cells were compared with the control (untreated cells) and expressed in percentage. The IC_50_ was then determined from the cell viability profile [28].

### 3.6. In Vivo Pharmacokinetic Studies Using Sprague Dawley Rats

Male Sprague Dawley (SD) rats (200 ± 20 g) were purchased from the BK Lab Animal, Ltd. (Shanghai, China) and housed at a temperature of 25 ± 1 °C and humidity of 50 ± 10% with free access to food and water. Three groups of SD rats (n = 6) were subjected to a single dose for oral bioavailability tests. The first group was intravenously injected with the gemcitabine solution at a dose of 80 mg/kg. The second group was administered the gemcitabine solution by oral gavage, while the third group received the drug-loaded NPs orally at the same dose [29]. Blood samples were drawn via retro-orbital venous plexus puncture with the aid of capillary tubes at predetermined intervals post-oral dose. The blood samples were collected in heparinized Eppendorf tubes, which were rinsed using 20 µm of tetrahydrouridine and then centrifuged at 42,018× *g* for 5 min; the plasma was then stored at −70 °C until analysis. An organic solvent was needed to extract gemcitabine from the plasma; to prepare the solvent, 1 mL of acetonitrile was added, vortexed for 1 min, and centrifuged at 101,890× *g* for 15 min. The supernatant was filtered and determined using HPLC. Prior to the animal studies, ethical approval was obtained by the Ethics Committee of Fudan University (Approval Number 04-YJ-LWY-01).

### 3.7. In Vivo Acute Toxicity Studies 

BALB/c nude mice were used for in vivo pharmacodynamic studies. In order to determine a suitable oral drug dose in this model, the LD_50_ had to be determined, as no data were available in the literature. Acute toxicity studies were conducted under ethics approval 04-YJ-LWY-01. Male BALB/c nude mice weighing 23 ± 2 g were purchased from BK Lab Animal, Ltd. (Shanghai, China) and housed in the same conditions as the SD rats. Five different doses of 60, 120, 240, 480, and 960 mg/kg were administered via oral gavage to each group (n = 10). Their motion behaviors were observed, weight loss and the number of deaths were recorded over 10 days, and the LD_50_ of BALB/c nude mice was determined [30].

### 3.8. In Vivo Pharmacodynamic Studies Using BALB/c Nude Mice

Three groups of BALB/c nude mice (n = 6) were used for in vivo pharmacodynamic studies. Prior to the study, 4T1 tumor cells were cultured at the same mentioned condition, and then the cells were collected and injected subcutaneously into the prep axillary (armpit) area of each mouse. Until the solid tumors were developed, the first group was orally administrated a saline solution, while the second and the third group were orally administrated the gemcitabine solution and gemcitabine-loaded NPs, respectively, at a dose of 30 mg/kg on days 0, 2, and 4. Tumor size and body weight were measured every 2 days throughout the study. The study was terminated after 12 days; the mice were euthanized, and the tumors were harvested.

### 3.9. Statistical Analysis 

Statistical data analysis was performed using the Microsoft Excel 2020 software (Redmond, WA, USA). Data comparisons were conducted using regression analysis and two-tailed *t*-tests. A *p*-value of ≤0.05 was considered statistically significant. 

## 4. Results and Discussion

### 4.1. Optimization of Polymeric Films, and Freezer Mill Settings

Polymeric films were formed with different polymer concentrations of 2%, 4%, 6%, and 8% *w*/*v*. As a result, the optimal films of MC, HPMC, and chitosan were more likely formed at lower concentrations, while EC and β-glucan with higher polymer concentrations formed more physically stable films. The water-soluble polymers MC, HPMC, β-glucan, and chitosan have high water absorbing and swelling properties; thus, they easily produce more viscous and gel-like forms, which allows for faster and better casting of the films [31,32]. Chitosan, β-glucan, and EC films generated fine and pulverized nanoparticulate powder after milling. However, for MC and HPMC formulations, the films were brittle and difficult to remove from the Petri dishes. This brittleness of MC and HPMC films is caused by their high intermolecular bonds between polymer chains [33]. Therefore, a small amount of polyethylene glycol (2% *w*/*v*) was added as a plasticizer. Plasticizer facilitates ease in film removal by reducing intermolecular interactions between these chains. The reduced inter-chain interaction also increases chain mobility and chain flexibility [31,33]. In contrast, the particles generated from the HPMC films did not produce pulverized powders as the HPMC NPs removed from the milling tubes were slightly moist and easily aggregated. This could be due to the high moisture retention properties of HPMC. HPMC films are known to have a poor water vapor barrier, which prevents water loss [34]. Due to clumping, characterization tests were not performed with HPMC NPs. The NPs generated from the other polymers were used in the next stage of characterization studies.

Although a previous study reported that extended milling times, normally over 10 min, resulted in progressively larger particle size, this study was performed using ball milling at room temperature. Physical milling is a high-energy process that may generate excess heat and can therefore cause alterations to the surface of the particles forming amorphous particle morphology. These areas are more likely to recrystallize, which may result in particle–particle fusion, thus increasing the particle size [35]. However, as our freezer mill operated at cryogenic temperatures, the effects of the high energy processes associated with milling may be less pronounced, thus leading to less particle aggregation. The optimal freezer mill settings were predicted based on the evaluation of the particles’ physical appearance and their particle size. The settings were 20 min precooling time, five operating cycles, and 10 min running/milling time with 14 CPS impact rate, followed by 5 min cooling time prior to the next milling cycle. 

### 4.2. Particle Size, Zeta Potential, Surface Morphology, and Entrapment Efficiency

A smaller particle size is more desirable, as there is a greater surface area of the NPs to adhere to the lining of the gut for drug absorption. Generally, there are three absorption mechanisms for NPs to permeate over the GI epithelial membrane [5]. Firstly, very small-sized particles of less than 50 nm often permeate the epithelial cells through tight junctions via the paracellular route. Particles of less than 500 nm are small enough to undergo endocytosis as a result of intestinal enterocytes lining the GI tract; the smaller the particles, the more efficient for the particles to undergo uptake through this route [5,36]. Particles less than 5 μm can be absorbed by the intestinal microfold cells of Peyer’s patches through the lymphatic system [37]. According to the results, β-glucan and EC NPs showed relatively smaller particle sizes of 447.6 ± 14.2 and 472.6 ± 21.7, respectively. Considering these sizes, it can be deduced that they are absorbed via the endocytosis pathway. 

Zeta potential is a measure of the magnitude of charge repulsion or attraction between particles, which is closely related to particle stability [38]. Good stability is important in preventing the aggregation of particles. In addition, zeta potential is an important parameter for investigating the interactive binding of NPs with the intestinal mucosal layer for absorption. NPs with zeta potential values of greater than +15 mV or less than −15 mV are considered to be stable, as they will repel each other and reduce the extent of aggregation [39]. The zeta potential results of the formulations are shown in Table 1. Although β-glucan is a cationic polymer, the zeta potential of the β-glucan NPs was −1.2 ± 0.5 mV. At low pH, it has a positive zeta potential, whereas at neutral or high pH, the zeta potential becomes negative. Since gemcitabine is negatively charged, it may have contributed to the negative zeta potential due to the adsorption of gemcitabine to the outer surface of the β-glucan NPs [40]. EC and MC NPs both exhibited negative zeta potentials, and EC NPs showed a desirable zeta potential of −20.1 mV. In contrast, chitosan NPs showed a positive surface charge. Through visual observation, some aggregation of β-glucan nanoparticles was observed, especially when resuspended in water. This was because the zeta potential results did not fall in the ideal range. This is one of the limitations that should be considered in future studies.

In the EE study, β-glucan showed the highest drug EE of 64.3 ± 2.1%, while EC had the lowest EE of 56.3 ± 1.8%. The reasons for the differences in drug EE between these formulations may be related to different interactions and the levels of these interactions between gemcitabine and the polymer chains [37]. The EE of NPs prepared using traditional methods such as solvent evaporation or solvent displacement is influenced by various factors such as the concentration of the polymer and emulsifier, drug-to-polymer ratio, and chemical interaction between the drug and polymers [37]. In contrast, the film casting method followed by cryogenic milling results in much higher EE than traditional methods, as the drug is fully dissolved into the polymer solution, and after the films are formed, it is directly ground into NPs. Thus, the drug is assumed to be greatly encapsulated due to being chemically bound to the polymer chains or physically embedded within the polymeric network of the particles. 

**Figure 1:**
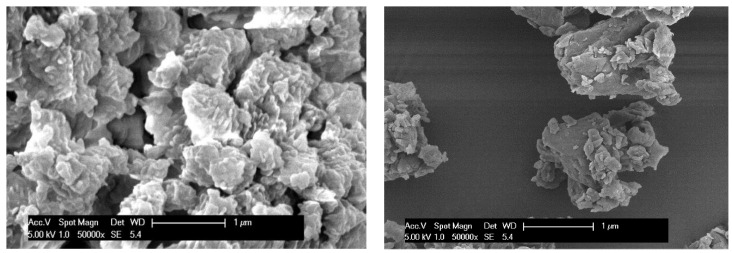
SEM images (50,000× magnification) of the optimal formulation of β-glucan NPs prepared via freezer milling.

Based on the results, β-glucan NPs were selected as the optimal formulation for further studies since they had relatively a smaller particle size, more promising zeta potential, and higher EE. As shown in the SEM images (Figure 1), β-glucan NPs have rough surfaces and irregular shapes; this is expected for particles fabricated using the freezer-milling method. 

### 4.3. In Vitro Drug Release Studies

**Figure 2:**
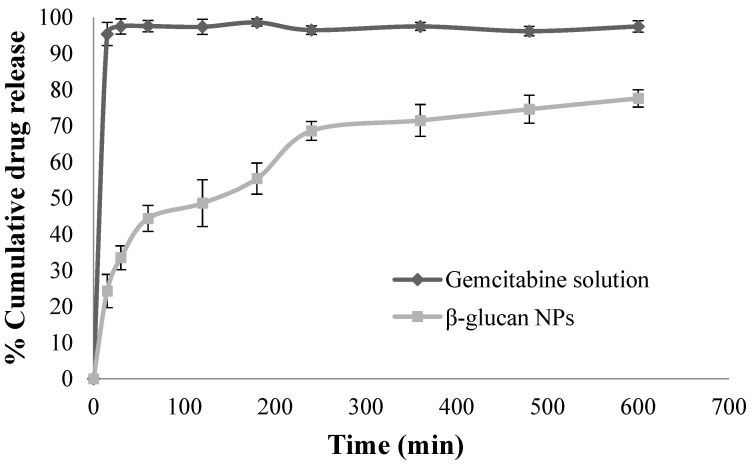
In vitro drug release of gemcitabine solution and gemcitabine loaded β-glucan NPs (mean ± SD, n = 3).

Through diffusion, the gemcitabine solution permeated the cellulose membrane with 95.4 ± 3.2% of the drug diffused into the receptor chamber within 15 min, while β-glucan NPs showed sustained release over 10 h (Figure 2). In fact, β-glucan NPs exhibited a biphasic drug release profile [41]. The first phase of the profile involved the release of unentrapped gemcitabine, which was adsorbed on the outer surface of the NPs, this was also considered to be an initial burst release. After this phase, a sustained release occurred, resulting from the slow diffusion of gemcitabine out of the polymer matrix [41]. The biphasic release profile suggested that the majority of the drug was entrapped within the polymer network rather than being adsorbed at the surface of the NPs [41]. Finally, the data obtained from the drug release were fitted to different kinetic models to investigate the kinetic drug release mechanisms. The modeled kinetic parameters are reported in Table 2. The release data were subjected to goodness-of-fit test (*r*^2^) using linear regression analysis according to the selected release kinetic models.

From the results, we can see that the Korsmeyer–Peppas model shows the highest *r*^2^ of 0.96, indicating that the gemcitabine release from β-glucan NPs complies with this model well. The diffusional release exponent (*n*) in the model can reflect the diffusion mechanism. When *n* = 0.45, Fickian diffusion is expected, in which diffusion is affected by the permeation of a solvent with constant velocity into the drug carriers. If *n* = 1.0, it demonstrates zero-order release kinetics, with release occurring independently of concentration. If 0.45 < *n* < 1, then it is non-Fickian or anomalous diffusion as a combination of Higuchi and zero-order diffusion methods [42]. The release exponent n was 0.52, indicating that the drug release was governed by diffusion through the NP matrix as well as matrix erosion, hence considered the so-called anomalous diffusion. This anomalous diffusion is evidence that the gemcitabine released from β-glucan NPs was controlled by more than one process.

This release profile demonstrates the benefit of β-glucan NPs in minimizing enzymatic degradation in the GI tract, ensuring the transition of most of the entrapped drug to the small intestinal membrane, where the main drug absorption occurs [43]. Moreover, the sustained release profile allows the intact drug-loaded NPs to retain in the systemic circulation for sufficient time, promoting the NPs to accumulate at the tumor site due to the EPR effect [44].

### 4.4. Ex Vivo Permeation Studies 

**Figure 3:**
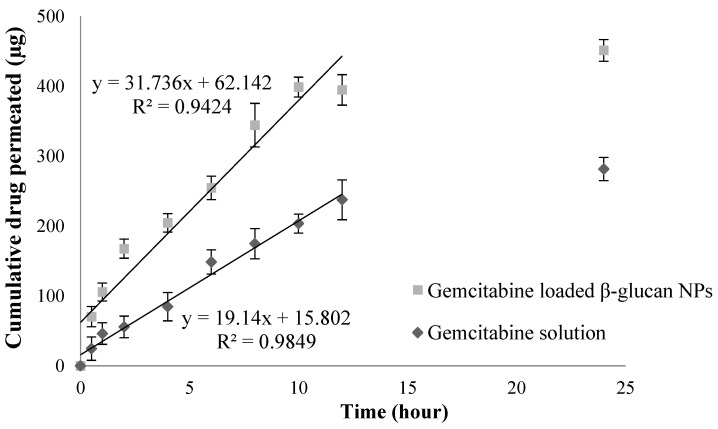
Ex vivo permeation studies of gemcitabine loaded β-glucan NPs and gemcitabine solution through the porcine intestinal epithelial membrane (mean *±* SD, n = 3).

The ex vivo permeation studies on the porcine intestinal epithelial membrane showed that the drug-loaded β-glucan NPs had a higher permeation rate than the plain drug solution (Figure 3). A total of 45.1 ± 3.8% gemcitabine was permeated through the epithelial membrane from the β-glucan NPs compared to 28.1 *±* 4.2% from the drug solution over 24 h. The results also showed that the flux of drug-loaded β-glucan NPs across the epithelial membrane was significantly higher than that of the control. The apparent partition coefficients (P_app_) were calculated using Equation (1). The flux (dX_r_/dt), which is the gradient of the best-fit line, was determined, and the results showed that drug-loaded β-glucan NPs and the drug solution had flux values of 31.74 and 19.14, respectively. The linear part of the permeation curve from time 0 to 12 h indicates that the main permeation process occurred during this period. The surface area *A* was 1.77 cm^2^, and *C*_0_ was 5000 µg/mL. The P_app_ of the drug solution and drug-loaded β-glucan NPs were determined as (2.17  ±  0.26) × 10^−3^ cm/s and (3.59  ±  0.22) × 10^−3^  cm/s, respectively (*p* < 0.05). The larger the P_app_ values, the higher the drug absorption, and thus the higher the oral bioavailability expected [45]; these results correspond to the results from the later pharmacokinetic studies.

### 4.5. Cell Culture and Cytotoxicity Studies

**Figure 4:**
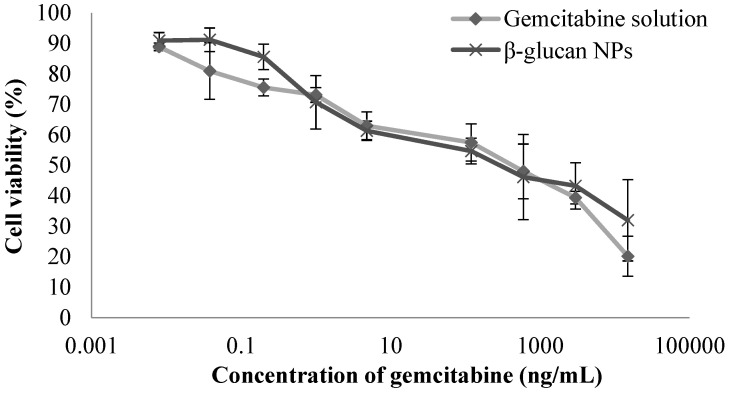
Cytotoxicity of gemcitabine solution and drug-loaded β-glucan NPs on 4T1 breast cancer cells at the tested concentrations (mean ± SD, n = 3).

Cytotoxicity studies for the gemcitabine solution and gemcitabine-loaded β-glucan NPs in 4T1 breast cancer cells were assessed based on mitochondrial activity (MTT) assay. As 4T1 tumor cells were used in the later pharacodynamic studies, the toxicity profiles of the formulations were evaluated before conducting the studies. The β-glucan polymer showed no significant cytotoxicity after 3 h of incubation with 4T1 cells, demonstrating the biocompatibility of the polymer with the tumor cells. Subsequently, the cytotoxicity studies of the drug solution and drug-loaded β-glucan NPs on 4T1 cells were performed for 72 h, and a dose-dependent cytotoxicity profile was observed (Figure 4). The IC_50_ values were then determined for the drug solution and drug-loaded β-glucan NPs, which were 228.8 ± 31.2 ng·mL^−1^ and 306.1 ± 46.3 ng·mL^−1^, respectively. Both the drug solution and the drug-loaded β-glucan NPs showed similar cytotoxicity profiles and similar IC_50_ values. In contrast, β-glucan NPs had slightly higher IC_50_ than the plain drug solution, which may be attributed to the fact that most of the drug was retained within the nanocarrier system with gradual drug release over time. 

### 4.6. In Vivo Pharmacokinetic Studies Using Sprague Dawley Rats

**Figure 5:**
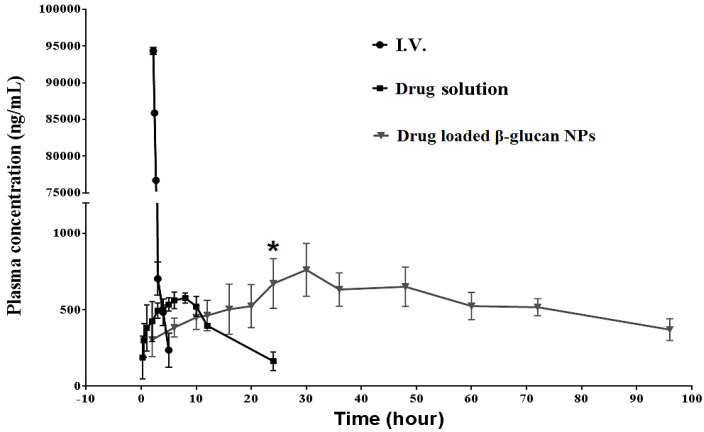
Plasma concentrations of the drug in SD rats following the oral administration of drug solution, drug-loaded β-glucan NPs, and *i.v*. injection of drug solution (mean ± SD, n = 6). The oral administration group results were significantly different from the results obtained with the *i.v*. administration of gemcitabine (*p* < 0.001) and the oral administration of gemcitabine solution; (*), *p* < 0.01.

In the pharmacokinetic study, the plasma drug concentration-versus-time profiles were plotted for the orally administered drug solution, drug-loaded β-glucan NPs, and intravenously (*i.v.*) administered drug solution in SD rats (n = 6), which are shown in Figure 5. Table 3 shows all the resulting pharmacokinetic parameters that were analyzed using GraphPad Prism Software (version 6.0). Veltkamp et al. (2008) found that the oral bioavailability of a pure gemcitabine solution was very low due to an extensive first-pass effect, but it was well tolerable at lower doses [46]. In our study, the maximum drug concentration (C_max_) of gemcitabine-loaded β-glucan NPs was 761.04 ± 214.32 ng/mL, which was higher than the plain drug solution of 577.11 ± 98.23 ng/mL. A higher C_max_ demonstrated the drug-loaded NPs were capable of bypassing hepatic first-pass metabolism and directly reaching the systemic circulation by virtue of the size and surface properties of the nanocarrier system [47]. The time at which C_max_ was reached (T_max_) for the drug solution and β-glucan NPs were 8 and 30 h, respectively. The delayed T_max_ indicated that, after intestinal transit, the majority of the drug was released while circulating in the systemic circulation. The area under the curve (AUC) value of drug-loaded β-glucan NPs was 46,457 ± 2124 ng·h/mL, significantly greater than that of the plain drug solution, which was only 9176 ± 785 ng·h/mL. The higher AUC of the drug-loaded β-glucan NPs indicated the sustained release of the drug candidate from NPs, which would avoid the drug being rapidly metabolised. Hao et al. reported a self-micro-emulsifying drug delivery system for the oral delivery of gemcitabine. Their developed carrier system enhanced the C_max_ significantly, but its half-life (T_1/2_) was not improved [48]. In this study, the T_1/2_ of drug-loaded β-glucan NPs was 69.98 ± 20.50 h, which was significantly improved compared with that of the pure drug solution at 9.40 ± 2.13 h. Finally, the absolute oral bioavailability of drug-loaded β-glucan NPs (49.92%) was 5.1-fold higher than that of the drug solution (9.86%).

### 4.7. In Vivo Acute Toxicity Studies

**Figure 6:**
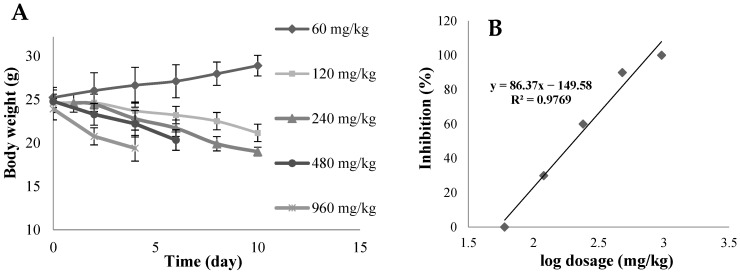
(**A**) Changes in the body weight of BALB/c nude mice after the oral administration of various dosages over 10 days (mean ± SD, n = 10); (**B**) percentage inhibition versus logarithm of dosages to determine LD_50_ of gemcitabine in BALB/c nude mice.

An in vivo acute toxicity study was required to determine a suitable oral dosage of gemcitabine for BALB/c nude mice prior to in vivo pharmacodynamic studies. Figure 6A displays the changes in the body weight of mice orally administered one of the five different dosages over 10 days. At doses above 60 mg/kg, gemcitabine caused a decline in body weight, and the higher the dosage, the larger the decrease in weight. At the highest dosage of 960 mg/kg, all mice died after 4 days, while 3, 6, 9, and 10 mice survived through to 10 days at 480 mg/kg, 240 mg/kg, 120 mg/kg, and 60 mg/kg dosages, respectively. Furthermore, over the 10 days of observation, the groups that received doses above 60 mg/kg suffered from diarrhea, with slower movement. Symptoms were more severe in groups administrated with higher doses of gemcitabine. Figure 6B shows the number of deaths plotted against log concentration. The LD_50_ was 204.17 mg/kg. 

### 4.8. In Vivo Pharmacodynamic Studies Using BALB/c Nude Mice

**Figure 7:**
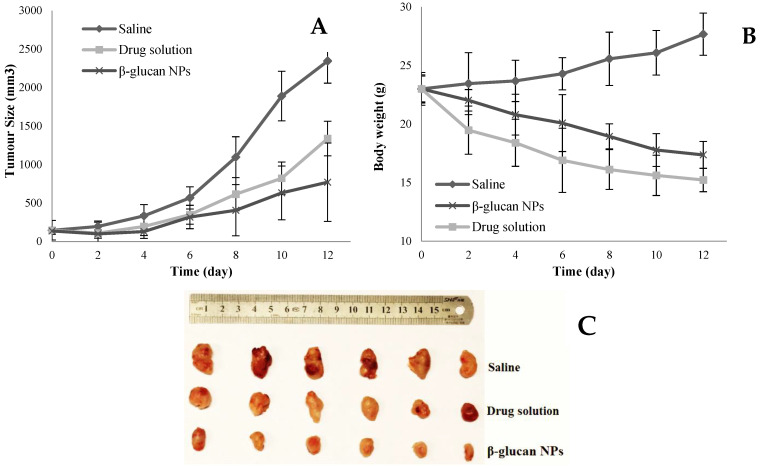
(**A**) The time course of tumor size in BALB/c nude mice recorded during the period of treatment with drug solution and drug-loaded β-glucan NPs, as well as in those without treatment (saline group) over 12 days (mean ± SD, n = 6, *p* < 0.01 at day 12); (**B**) the body weight change in BALB/c nude mice during the period of treatment over 12 days (mean ± SD, n = 6, *p* < 0.01 at day 12); (**C**) photography of the solid tumors with saline, 3 doses of 30 mg/kg drug solution, and an equivalent dose of drug-loaded β-glucan NPs, given on days 0, 2, and 4, and harvested on day 12 (n = 6).

The size of 4T1 breast tumors in the control group with no treatment markedly increased over the study period, while the tumor growth rate significantly reduced (*p* < 0.01) in the drug-loaded β-glucan NP group, with 3.04-fold and 1.74-fold reduction compared to the untreated control group and the drug solution group, respectively. This result corresponds to the in vivo pharmacokinetic studies indicating greater oral bioavailability and longer plasma half-life of drug-loaded β-glucan NP group compared to the plain drug solution group. Figure 7B shows a significant reduction in body weight of nude mice in both the drug solution group and the drug-loaded β-glucan NP group. The drug solution group had the greatest body weight loss, which demonstrated the high toxicity in this group. This phenomenon also corresponds to the acute toxicity study, which revealed that the mice in the drug solution group had severe diarrhea, reflecting the serious local side effects induced in the GI tract [49]. Interestingly, despite the greatly elevated oral bioavailability of the drug-loaded β-glucan NPs, a lesser reduction in body weight was observed. This may suggest that β-glucan NPs are absorbed into the systemic circulation as intact NPs [43,47,50], as well as limited drug exposure to nontarget organs. 

## 5. Conclusions

Gemcitabine-loaded polymeric films were prepared using the conventional casting method, and then pulverized NPs were obtained via the freezer-milling technique. The NPs showed promising characteristics of a relatively small particle size of 447.6 ± 14.2 nm, a relatively high entrapment efficiency of 64.3 ± 2.1%, a sustained in vitro drug release profile, and greater ex vivo permeation through the porcine intestinal epithelial membrane compared to the plain drug solution. Cytotoxicity studies highlighted the safety of the β-glucan polymers, and the IC_50_ values of the gemcitabine solution and gemcitabine-loaded β-glucan NPs were also determined. Pharmacokinetic studies in SD rats showed that gemcitabine-loaded β-glucan NPs achieved a 7.4-fold longer T_1/2_, and a 5.1-fold increase in absolute oral bioavailability was observed compared with plain drug solution. Furthermore, in vivo pharmacodynamic studies showed the promising capability of gemcitabine-loaded β-glucan NPs to inhibit 4T1 breast tumor growth, suggesting that this novel freezer-milled β-glucan NP system is a suitable drug delivery strategy for the oral delivery of gemcitabine and a promising potential platform for oral chemotherapy. 

## Figures and Tables

**Table 1:** Particle size, PDI, zeta potential, and EE of drug-loaded freezer-milled NPs (n = 3, mean ± SD).

	Particle Size (d.nm)	PdI	Zeta Potential (mV)	Entrapment Efficiency (%)
MC NPs	555.0 ± 32.2	0.24 ± 0.13	−0.6 ± 0.7	63.9 ± 1.8
β-Glucan NPs	447.6 ± 14.2	0.18 ± 0.07	−1.2 ± 0.5	64.3 ± 2.1
EC NPs	472.6 ± 21.7	0.32 ± 0.11	−20.1 ± 1.2	56.3 ± 1.8
Chitosan NPs	643 ± 52.4	0.13 ± 0.11	+6.9 ± 1.4	60.2 ± 1.3

**Table 2:** Release kinetic parameters of β-glucan NPs subjected to different drug release models.

	Zero Order		First Order		Higuchi Model		Korsmeyer–Peppas Model		
β-Glucan NPs	*r* ^2^	*k*_0_	*r* ^2^	*k*_1_	*r* ^2^	*k_h_*	*r* ^2^	*n*	*k_k_*
	0.73	28.45	0.88	1.86	0.94	20.75	0.96	0.52	1.04

**Table 3:** Pharmacokinetic parameters of gemcitabine in SD rats following the oral administration of drug solution, drug-loaded β-glucan NPs, and *i.v.* administration of drug solution (mean ± SD, n = 6).

PK Parameters	Gemcitabine (*i.v*.)	Gemcitabine Solution (Oral)	β-Glucan NPs (Oral)
C_max_ (ng/mL)	94,152 ± 3435	577.11 ± 98.23	761.04 ± 214.32
T_max_ (h)	0.25	8	30
AUC_0-inf_ (ng·h/mL)	93,050 ± 1459	9176 ± 785	46,457 ± 2124
T_1/2_ (h)	1.18 ± 0.85	9.40 ± 2.13	69.98 ± 20.50
Oral bioavailability	100%	9.86%	49.92%

## Data Availability

All the data is contained within the article.
